# A multilevel examination of the association between COVID-19 restrictions and residence-to-crime distance

**DOI:** 10.1186/s40163-022-00172-1

**Published:** 2022-11-14

**Authors:** Theodore S. Lentz, Rebecca Headley Konkel, Hailey Gallagher, Dominick Ratkowski

**Affiliations:** 1 Department of Criminal Justice and Criminology, University of Wisconsin-Milwaukee, 2400 E. Hartford Avenue, Milwaukee, WI 53211 USA; 2 Wauwatosa Police Department, Wauwatosa, USA

## Abstract

Restrictions resulting from the COVID-19 pandemic interrupted people’s daily routine activities. Rooted in crime pattern and routine activity theories, this study tests whether the enactment of a Safer-at-Home mandate was associated with changes in the distance between individuals’ home addresses and the locations of where they committed crimes (i.e., residence-to-crime distance). Analyses are based on violent (N = 282), property (N = 1552), and disorder crimes (N = 1092) reported to one police department located in a United States’ Midwest suburb. Multilevel models show that residence-to-crime distances were significantly shorter during the Safer-at-Home order, compared to the pre- and post-Safer-at-Home timeframes, while controlling for individual and neighborhood characteristics. Additionally, these relationships varied by crime type. Consistent with the literature, the findings support the argument that individuals tend to offend relatively near their home address. The current findings extend the state of the literature by highlighting how disruptions to daily routine activities stemming from the COVID-19 pandemic led to alterations in crime patterns, in which analyses indicated shorter distances between home address and offense locations.

**Supplementary Information:**

The online version contains supplementary material available at 10.1186/s40163-022-00172-1.

## Introduction

Environmental criminology is concerned with how environmental context shapes the convergence of potential offenders and crime targets (Brantingham & Brantingham, 1993; Cohen & Felson, 1979). One specific focus in this literature is the process of searching for crime targets (e.g., offender foraging perspective; Vandeviver et al., 2021), including the route from an individual’s home to the site of the crime event, loosely referred to as the “journey to crime” (Rengert, 2002). Several factors are believed to affect one’s choice of where to offend, such as the spatial distribution of target suitability and perceived risk factors (Clarke & Cornish, 1985), one’s individual mobility constraints (e.g., lack of public transportation), and broader routine activity patterns (Brantingham & Brantingham, 1993; Cohen & Felson, 1979). Additionally, the literature suggests that one’s home address is the location from which individuals begin their journey to crime (for example, Bernasco, 2006; Rengert & Lockwood, 2009; Van Daele et al., 2012). We use this perspective to frame the current study, which examines whether abrupt social changes stemming from the COVID-19 pandemic affected the residence-to-crime distance observed in a United States suburban setting. Specifically, we test for changes in the average distance between an individual’s home and the site of their crime during when “Safer-at-Home” order was implemented, while controlling for individual and neighborhood factors. Although recent studies have examined the relationship between COVID-19 and crime frequencies and rates, no work has examined associations between social distancing restrictions and journey to crime patterns. Findings from this study may be helpful to communities and law enforcement in preparing for and responding to future social changes that may similarly affect crime patterning.

The current study is framed in routine activity theory (“RAT”) and crime pattern theory. Cohen and Felson’s (1979) RAT argues that crimes are most likely to occur when a motivated offender, suitable target, and lack of a capable guardian converge in space and time. Specifically, they argue that the temporal and spatial juncture of these elements are heavily dependent on daily mobility patterns (e.g., traveling to/from work, social arenas, place of residence). Building on this framework, more recent research has been supportive of the crucial role that routine activity patterns play in the formation of crime patterns (Browning et al., 2021; Song et al., 2019). Further, research shows that disruptions to the daily routines of a spatial area, such as an influx in the number of visitors, impacts the spatial patterning of crime (Boivin & Felson, 2018).

Relatedly, crime pattern theory offers a more nuanced explanation of how criminal opportunities are influenced by the places where an individual goes in their daily life. Brantingham and Brantingham (1981) discuss the intertwined relationship between where one resides (“anchor”), spend time socializing, working, and carrying out other activities (“nodes”), and the routes taken between the anchor and other nodes (“pathways”). The geographical area encapsulating anchors, other nodes, and pathways is called “activity space.” People are most familiar with this area and are better able to read its environmental and social cues (Menting et al., 2019); consequently, it is the area where they are most likely to offend (Curtis-Ham et al., 2020; Lammers, 2018). Due to the COVID-19 restrictions that resulted in vast closures of nodes outside of one’s anchor, one would expect individuals to offend closer to their homes, as many nodes and pathways to these nodes were, presumably, less frequented while social distancing mandates were in place.

Many factors can affect the distance between an individual’s home and the location of their respective offense. Studies have indicated that individuals tend to offend near their residences (often within 1 or 2 miles; Bernasco, 2006; Hammond & Youngs, 2011; Rengert & Lockwood, 2009; Van Daele et al., 2012), although distance traveled often varies by crime type (Bernasco & Block, 2009; Bernasco & Nieuwbeerta, 2005; Elffers et al., 2008; Hammond & Youngs, 2011; Menting et al., 2019; O’Leary, 2011; Van Daele, 2010). For example, violent crimes typically occur closer to one’s home than property crimes (Ackerman & Rossmo, 2015). Beyond crime type, individual characteristics also affect the distance one travels to commit crime. In one study, Andresen and colleagues (2014) found that the distance between an individual’s residence and the location of their crime was influenced by a quadratic function of individual age. Specifically, age had a decaying quadratic effect on distance between one’s residence and the location of their offense, in which age was positively associated with distance traveled until age 20, and then this association became inversive (Andresen et al., 2014; also see Ackerman & Rossmo, 2015). Other findings suggest that individuals are influenced by the presence of specific crime attractors that can “pull” them further from their home to engage in crime (Bernasco & Block, 2009). Taken together, existing research demonstrates that one's journey to crime is affected not just by individual characteristics, but also by the larger environmental context in which crimes occur.

The COVID-19 pandemic and efforts implemented to stop the spread of the virus have had a profound effect on social and environmental contexts. Through the enactment of policies promoting social distancing, such as curfews, closing of non-essential businesses (e.g., bars, restaurants), and encouraging people to stay home, many individuals’ daily routines and travel patterns were presumably altered. In this light, we anticipate that disruptions in daily activities brought on by Safer-at-Home orders would impact the location of offenses due to decreased access to non-anchor nodes. It is possible, however, for this relationship to be in either direction. For example, because individuals were mandated to stay at home, the likelihood that motivated offenders and suitable targets meet in public spaces should decrease, but public guardianship may also be at a lower level. On the other hand, offenses that generally take place within the home (e.g., domestic offenses) may increase, as offenders and victims may be spending more time together. Conversely, offenses that take place against one’s home (e.g., residential burglary) would likely be hypothesized to decrease, as residential guardianship level would be higher.

Emerging research supports the notion that COVID-19 restrictions disrupted pre-pandemic crime levels, as well as spatial and temporal crime patterns (Halford et al., 2020; Nivette et al., 2021). Researchers have consistently found that crime rates were impacted by “Shelter-in-Place” (e.g., Safer-at-Home) orders, however, the direction and size of these effects vary by crime type. For example, some studies observed reductions in residential burglaries, robberies, rapes, assaults, and thefts (Ceccato et al., 2022; Felson et al., 2020; Mohler et al., 2020), whereas domestic violence and nonresidential burglaries were found to increase (Ceccato et al., 2022). Other research has found that Safer-at-Home orders resulted in travel pattern disruptions, decreased attendance to heavily trafficked attractions (Yang et al., 2021), and lower levels of guardianship in non-residential areas (Campedelli et al., 2020; Felson et al., 2020; Mohler et al., 2020). Consequently, findings point to dramatic shifts in the locations of traditionally high and low crime areas (Campedelli et al., 2020; Ceccato et al., 2022), as the locations of guardians and targets migrated from public to private/residential areas (Yang et al., 2021).

While environmental factors have been considered in past research, there is limited research on the association between COVID-19 changes and the distance one travels from home to commit crimes. The current study takes nuanced approach by examining the relationship between COVID-19-related changes and the distance from one’s home to the location of their offense. Additionally, analyses test whether the association varies by crime type.

## Methods

### Study suburb

Crime data for this study came from one police department located in a Midwestern suburb in the United States. The jurisdiction has a population of 48,000 residents and spans 13.2 square miles within the state’s most populous county. The sample suburb has an average violent crime rate of approximately 3.6 per 1000 residents, but the city directly adjacent is among the most violent in the U.S. with a violent crime rate of approximately 13.5 per 1000 residents. Conversely, the study suburb has a slightly higher property crime rate (28.8 per 1000 residents) than does the adjacent city (26.3 per 1000).

The statewide Safer-at-Home order mandated that all individuals must stay in their place of residence, except for carrying out essential activities, which were limited to activities related to health and safety, procuring necessary supplies or services, outdoor activities while social distancing, performing essential types of work, and providing care to other individuals or pets). Given that human movement, social gatherings, and other “routine activities” (e.g., non-essential work, school, travel, entertainment, non-essential services) were greatly impacted by Safer-at-Home orders, crimes occurring in or near suburban areas are particularly likely to be affected (for complete list of all restrictions during the Safer-at-Home order, see https://evers.wi.gov/Documents/COVID19/EMO12-SaferAtHome.pdf).

The study suburb is advantageous for multiple reasons. First, findings from this study may highlight important contextual features of crime patterns, given that 69% of the crimes in the sample were committed by individuals whose home addresses were located outside of the study jurisdiction. Secondly, suburban areas tend to have fewer public transportation options, which implies that residents might have to travel longer distances to engage in routine activities. Third, criminological studies largely focus on urban areas, with more recent attempts to include rural settings. Few studies have considered the “middle-ground” of suburbia. Because of spatial, economic, and ideological differences between urban, suburban, and rural populations, findings from past urban and rural studies may not be generalizable to suburban areas (Nivette et al., 2021).

### Data

Data for this study come from two sources. Data on criminal offenses and characteristics of individuals who have been arrested for each respective offense come from the study suburb’s police department. Hereafter, we use person-centered language and refer to those in our sample simply as “individuals” to avoid labeling them as “offenders,” especially given that they may not have been charged and convicted of the offense for which they were arrested.

Secondly, neighborhood sociostructural data were obtained from the United States Census Bureau’s American Community Survey (2019, 5-year estimates) for all block groups in the sample jurisdiction and the surrounding 20-mile radius (N = 1210 block groups). Following past research (Ackerman & Rossmo, 2015), we chose the block group as our spatial grouping variable because it is the smallest spatial unit for which income data are readily available from the census. Block groups maximize between-area variation and minimize within-area variation of model factors.

### Measures

#### Dependent variable

The distance between an individual’s home and the location of their crime is used as the dependent variable and referred to as the “residence-to-crime distance”. To construct this variable, crime incident and individual data were collected on all offenses that occurred within the study suburb between January 1, 2018 and December 31, 2020. Crime incident data included the date, time, location, and offense type for each incident. Incidents involving multiple individuals were treated as separate crimes to allow each individual to represent a unique “residence-to-crime distance” pathway. Using a geographic information system (ArcPro 2.6), offense addresses were geolocated, and Euclidean (i.e., “as the crow flies”) distance analysis was used to calculate the distance in miles between each individual’s home address and the location of their respective offense (see Ackerman & Rossmo, 2015; Andresen et al., 2014; Menting et al., 2019; Xiao et al., 2018). Cases that were missing data on the location of the offense or home address (N = 11) or individual demographics (N = 7) were removed omitted from the dataset. All remaining cases were geocoded with 100% success by utilizing a composite geolocator (based on Esri’s geolocator tool and a dual ranges geolocator built for the sample city). Of the remaining cases, five were found to have residence-to-crime distances that were greater than 20 miles, which were omitted from the dataset to minimize outliers, and subsequently skewed and erroneous results.^1^ The final sample yielded N = 2926 offenses.

#### Independent variables

##### Block group-level variables

Block-group characteristics were used as a proxy for neighborhood context in two ways. For each offense, block group characteristics were recorded for (1) the individual’s home block group, and (2) the block group where the offense occurred. The former set of block group variables measures the neighborhood context in which the individual lived, whereas the latter reflects the neighborhood context where the crime occurred.

Based on the literature, several neighborhood-level variables associated with the locations of crimes were included in analysis (for example, Bursik & Grasmick, 1993; Peterson et al., 2000; Sampson & Groves, 1989; Sampson et al., 2002; Shaw & McKay, 1942). Specifically, two variables were created to examine the relationship between neighborhood socioeconomic context and the residence-to-crime distance, including concentrated poverty and residential instability. Based on Massey’s (2001) index of concentrated extremes, *concentrated poverty* was captured using the following equation:

1 $$\mathrm{Concentrated\; poverty}=\left[\frac{(\mathrm{Number\; of\; affluent\; housholds}-\mathrm{Number\; of\; impoverished\; households})}{\mathrm{Total \;number\; of \;households}}\right]$$

This produced an index where a value of − 1 indicates all households had annual earnings of at least $100,000, and a value of + 1 indicates extreme concentrated poverty, in which all households earning less than $25,000 annually.^2^*Residential instability* was captured by a factor score based on a principal components factor analysis with a varimax rotation including (1) the percent of residents who reported moving into their current home within the past 5 years, and (2) the percent of renter occupied housing units (alpha = 0.749; Eigenvalue = 1.737; all factor loadings > 0.932).

Based on Simpson’s (1949) and Blau’s (1977) diversity and heterogeneity indexes, a measure reflecting *racial diversity* was created. This measure was computed as 1 − ∑(p_*i*_^2^), where p_*i*_ is the proportion of each racial category within the population (see also Cahill & Mulligan, 2007). This index ranges from 0 to 1, where 1 indicates maximum racial diversity and 0 indicates maximum racial homogeneity. In addition, the percentage of neighborhood residents who were males between 15 and 24 years old (“*percent young males*”), and *population density* (residents/square mile) were included within models to account for high-risk areas.

##### Individual-level variables

To examine whether residence-to-crime distances were affected by a Safer-at-Home order in place between March 25 and May 13, 2020, offenses were disaggregated into three categories, including those that occurred (1) prior to the start of the Safer-at-Home order (*Pre-Safer-at-Home*; N = 2340),^3^ (2) during the Safer-at-Home order (*During-Safer-at-Home*; N = 84),^4^ and (3) after the disbandment of the Safer-at-Home order (*Post-Safer-at-Home*; N = 502).^5^ Additionally, to examine whether the residence-to-crime distance varied by type of offense, offenses were categorized as *violent offenses* (i.e., robbery, battery, reference category; N = 282), *property offenses* (i.e., burglary, theft, arson; N = 1552), or *disorder offenses* (i.e., disorderly conduct, gambling, indecent exposure, loitering, narcotics, ordinance violations, trespassing, vandalism, suspicious behavior; N = 1092).^6^

Several key demographic indicators were also entered into models as controls and included the individual’s sex (1 = *male*; 0 = female), *age* (i.e., under 18 years of age, 18 to 24 years of age, 25 to 34 years of age, or 35 years of age or older), and *race* [i.e., American Indian/Alaskan Native, Asian, Black, White). Lastly, to account for seasonal effects related to offending (Castle & Kovacs, 2021), a series of *month* dummy variables were included in each model.

### Analytic strategy

As part of the descriptive analysis, least squares analysis of variance was employed to identify significant differences in the mean residence-to-crime distance along key independent measures. Next, multilevel modeling was used to examine whether the enactment and abandonment of the Safer-at-Home order was associated with residence-to-crime distances, while controlling for individual and neighborhood characteristics. Individuals living spatially close to one another are often exposed to similar environments, and thus, expected to have more similar behavioral outcomes than those living in different environments. Because this can lead to non-independence of observations and error terms, we employed multilevel modeling to account for the nested nature of these data, and to allow for individual- and neighborhood-level variables to be included within one parsimonious model (Raudenbush & Bryk, 2002).

Using statistical software (Stata 15.1), a series of multilevel linear models were estimated based on the following formula:

$${\mathrm{Level}}\,1{:} \quad {Y_{ij}}={\beta }_{0j}+{\beta }_{kj}{{\varvec{X}}}_{k}$$

$$\mathrm{Level }\,2{:} \quad {\beta }_{0j}={\gamma }_{00}+{\gamma }_{0m}{{\varvec{W}}}_{m}+{u}_{0j}$$

In the level 1 equation, *Y*_*ij*_ represents the square root of the distance between individual *i*’s residence in block group *j* to *i*’s corresponding crime location. The *β*_*0j*_ coefficient represents the average probability across block groups, which is modeled at level 2. In the level 1 equation, the *β*_*kj*_***X***_*k*_ parameter represents a vector of individual level covariates along with their regression coefficients. The error term *u*_*0j*_ represents between block group variability in the residence-to-crime distance. The model intercept at level 2 is *γ*_*00*_ and *γ*_*0m*_***W***_*m*_ is a vector of block group covariates used to explain neighborhood variation in the residence-to-crime distance. Additionally, month random effects were included to adjust for seasonality effects related to routine activity behaviors and crime patterns. Separate models were estimated to assess differences across broad offense types (i.e., violent, property, disorder offenses).

Studying the residence-to-crime distance presents unique challenges for multilevel modeling. The offenses in our data are nested in several ways, including within individuals (repeat offending), within incidents (co-offending), and within block groups (spatial autocorrelation). From an analytic standpoint, it is not feasible to incorporate all sources of clustering simultaneously into one model (multiple levels of cross-nesting). Given our focus on residence-to-crime *distance* and hypothesized importance of *place*, we chose to nest offenses in the block groups (geographic places) where the offense occurred (hereafter *crime block group*) and where the individual lives who was arrested for the crime (hereafter *home block group*). To adjust for these sources of spatial cross-nesting, cross-classified mixed effects regression models were estimated to account for multiple membership in two level-2 contexts (Johnson, 2012). Thus, our final sample of N = 2926 offenses were committed in 67 unique block groups by individuals with home locations in 668 unique block groups.

Following past research (Ackerman & Rossmo, 2015), a square root transformation of the residence-to-crime distance was used to reduce positive skewness in the distribution and improve model fitness. Consequently, the regression coefficients represent the difference between the square root of the distance for a given category of the independent variable (e.g., pre-Safer-at-Home) and the square root of the distance for the reference category (e.g., during Safer-at-Home) for the average individual. To make the results more interpretable, expected distance values were calculated by squaring the sum of the constant and the corresponding regression coefficient for a given variable, which resulted in the model producing estimates closer to the conditional median than to the conditional mean (Ackerman & Rossmo, 2015).

## Results

**Fig. 1:**
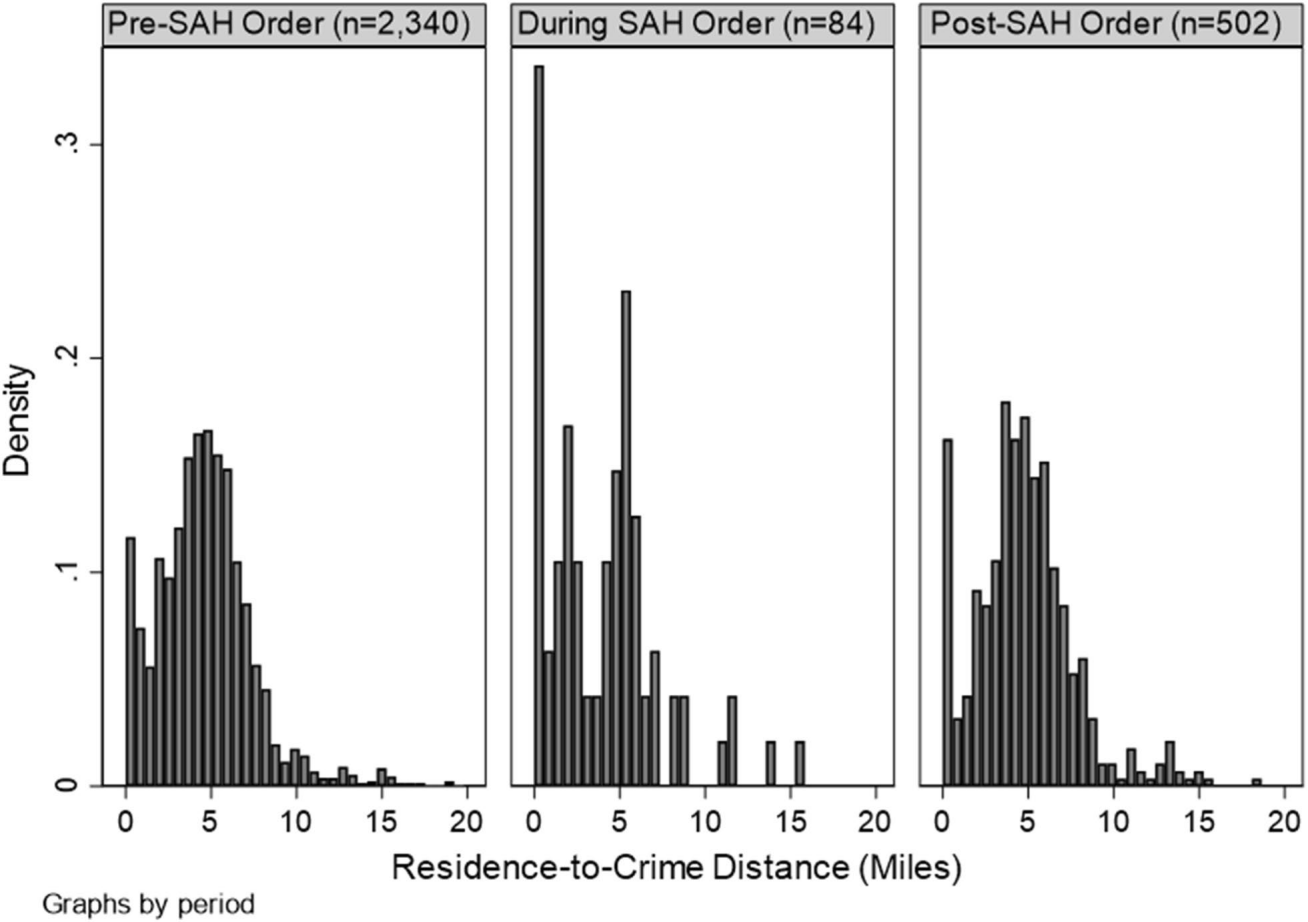
Histograms showing residence-to-crime distance in miles for crimes occurring in a Midwest suburb before, during, and after a Stay-at-Home order was enacted (2018–2020). Densities, rather than frequencies, are shown for comparison

**Fig. 2:**
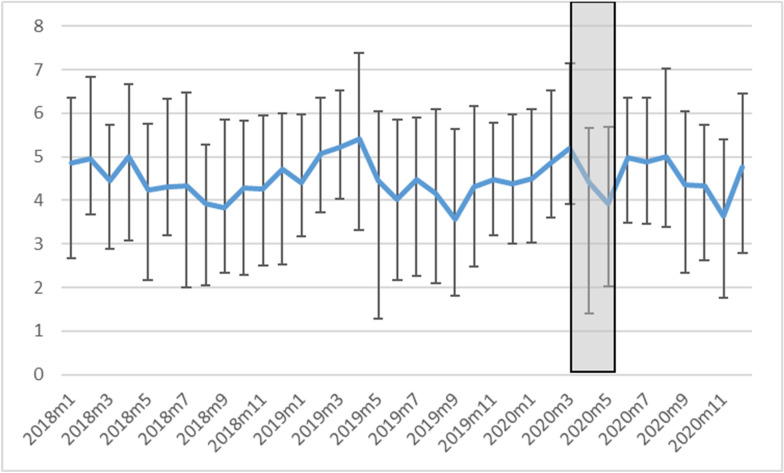
Monthly changes in median residence-to-crime distance (line chart; left axis; error bars indicate 25th and 7th percentiles) for N = 2926 crimes occurring in Midwest suburb (2018–2020). The Safer-at-Home order was in place March 25, 2020 through May 13, 2020, illustrated by the grey-shaded box

Table 1 reports summary statistics for all variables included in analysis. Throughout the 3-year study period, the average residence-to-crime distance was 4.64 miles (SD = 2.9; see distribution in Fig. 1). Figure 2 shows monthly changes in the total number of crimes that occurred and the mean residence-to-crime distance. Although inconclusive, we observed an unprecedented decrease in the mean residence-to-crime distance during April 2020, which is the time directly following the enactment of the Safer-at-Home order (March 25–May 13).

**Table 1:** Summary statistics for independent variables used in analysis

Variable	Mean/Proportion	SD	Median	25th PCTL	75th PCTL
Dependent variable (N = 2926)					
Residence-to-crime distance	4.640	2.900	4.511	2.780	6.104
Level 1 (N = 2926)					
Time period					
Pre-Safer-at-Home	0.800	–	–	–	–
During Safer-at-Home	0.029	–	–	–	–
Post-Safer-at-Home	0.171	–	–	–	–
Offense type					
Violent crime	0.096	–	–	–	–
Property crime	0.530	–	–	–	–
Disorder crime	0.373	–	–	–	–
Age					
0–17	0.055	–	–	–	–
18–24	0.344	–	–	–	–
25–34	0.266	–	–	–	–
35+	0.335	–	–	–	–
Sex					
Male	0.664	–	–	–	–
Female	0.336	–	–	–	–
Race					
AIAN	0.002	–	–	–	–
Asian	0.011	–	–	–	–
Black	0.732	–	–	–	–
White	0.254	–	–	–	–
Month					
Jan	0.094	–	–	–	–
Feb	0.080	–	–	–	–
Mar	0.076	–	–	–	–
Apr	0.070	–	–	–	–
May	0.068	–	–	–	–
June	0.088	–	–	–	–
July	0.083	–	–	–	–
Aug	0.090	–	–	–	–
Sep	0.088	–	–	–	–
Oct	0.086	–	–	–	–
Nov	0.076	–	–	–	–
Dec	0.101	–	–	–	–
Level 2 (N = 1120 block groups)					
Population density	6605.732	6058.899	5156.784	1893.916	9563.363
% Young males	6.559	5.204	5.551	3.231	8.599
Concentrated poverty	− 0.043	0.350	− 0.055	− 0.307	0.216
Residential instability	0.000	1.000	− 0.0850	− 0.849	0.763
Racial diversity	0.258	0.181	0.224	0.105	0.399

Table 2 reports residence-to-crime distance comparisons along several categorical distinctions. Prior to the Safer-at-Home order, the average residence-to-crime distance was 4.64 miles (SD = 2.86). During the Safer-at-Home order, the average distance decreased by approximately 15% (3.99 miles; SD = 3.34), and then increased by approximately 20% (4.77 miles; SD = 2.97) after the restriction was disbanded, which was slightly longer than the distance prior to the Safer-at-Home order. The model F-statistic suggests these distances were significantly different (p = .005). Beyond temporal differences, significant differences were observed in the residence-to-crime distance by offense type, as well as by the individual’s reported age, sex, and race.

**Table 2:** Residence-to-crime distance comparisons

Variable	N	Mean	Median	SD	Min	Max	*F*^+^	*p*
Time period							5.25	.0053
Pre-safer at home	2340	4.64	4.51	2.86	0.00	19.21		
During safer at home	84	3.99	4.07	3.34	0.00	15.66		
Post-safer at home	502	4.77	4.59	2.97	0.00	18.35		
Offense type							145.80	< .0001
Violent crime	282	3.60	3.64	3.03	0.00	18.66		
Property crime	1552	5.29	5.13	2.64	0.00	19.21		
Disorder crime	1092	4.00	3.75	2.99	0.00	18.95		
Age							9.98	< .0001
0–17	161	3.54	3.57	2.28	0.00	15.08		
18–24	1006	4.35	4.28	2.66	0.00	18.95		
25–34	778	4.99	4.74	3.03	0.00	19.21		
35+	981	4.85	4.84	3.04	0.00	18.66		
Sex							25.48	< .0001
Male	1944	4.47	4.31	2.89	0.00	18.95		
Female	982	5.00	4.85	2.88	0.00	19.21		
Race							13.67	< .0001
AIAN	7	5.21	4.50	2.27	2.96	9.24		
Asian	32	3.31	2.85	2.97	0.00	9.66		
Black	2143	4.60	4.59	2.24	0.00	16.91		
White	744	4.83	4.04	4.25	0.00	19.21		
Total	2926	4.64	4.51	2.90	0.00	19.21		

+Model statistic for analysis of variance (*df* = 2925)

*AIAN* American Indian-Alaskan Native

Next, intraclass correlation coefficients (ICCs) within null models were examined prior to full multilevel modeling. ICCs indicated that the amount of variation in distance traveled that was explained at level 2 was much greater when modeling home neighborhoods (ICC = 0.830) than crime location neighborhoods (ICC = 0.214); however, a substantial amount of variation in the outcome could be explained in both models at level 1 and level 2. Therefore, we proceeded to conduct full multilevel models to control for the effects of individual and neighborhood factors on the residence-to-crime distance.

Table 3 reports results from the regression models estimating associations of individual and neighborhood level covariates on the residence-to-crime distance for all offenses. Model 1 only included individual level characteristics and showed that both the pre- and post-Safer-at-Home periods had significantly longer residence-to-crime distances compared to during the Safer-at-Home timeframe. Recall that the dependent variable is a square root transformation and results are not readily interpretable; however, coefficients were converted to expected distances to improve interpretability of the results. For example, while controlling for all other level 1 variables, the average residence-to-crime distance for crimes committed prior to the Safer-at-Home order was 2.15 miles [(1.190 + 0.278)^2^ = 2.15], whereas during the Safer-at-Home order the average residence-to-crime distance was 1.41 miles. This implies a statistically significant 0.74 mile decrease in the average residence-to-crime distance during the Safer-at-Home order (p = .001), independent of the other predictors in the model. On average, property offenses were committed 3.16 miles further from one’s home than were violent offenses (p < .001), and disorder offenses were committed 2.00 miles further from one’s home than violent offenses (p < .001). Regarding individual characteristics, older individuals tended to commit crimes further from their homes, compared to those 17 years of age or younger. On average, individuals who identified as Asian committed crimes significantly closer to their homes (*b* = − .406; p = .002), whereas those who identified as Black committed crimes significantly further from their homes (*b* = .125; p < .001), compared to those who identified as White. Lastly, males committed crimes closer to their homes compared to females on average (*b* = − .079; p = .006).

**Table 3:** Regression models examining factors associated with residence-to-crime distance (N = 2926 Crimes)

Variable	Model 1	Model 2^+^	Model 3^++^	Model 4^+++^
Level 1				
Time period (ref. = During)				
Pre-safer at home	0.278**	0.211***	0.195*	0.140***
Post-safer at home	0.372***	0.229***	0.248**	0.123**
Offense type (ref. = Violent)				
Property	0.588***	0.232***	0.415***	0.144***
Disorder	0.225***	0.109***	0.228***	0.113***
Age (ref. = 0–17 years)				
18–24	0.137*	0.048	0.078	0.014
25–34	0.245***	0.037	0.155*	− 0.009
35+	0.166**	− 0.012	0.087	− 0.055*
Male (ref. = Female)	− 0.079**	− 0.055***	− 0.004	− 0.001
Race (ref. = White)				
AIAN	0.137	0.091	0.198	0.086
Asian	− 0.406**	0.048	− 0.344**	0.115
Black	0.125***	− 0.051*	0.073*	− 0.031
Month (ref. = Jan)				
Feb	0.120	0.027	0.088	0.003
Mar	0.103	− 0.013	0.102	− 0.014
Apr	0.032	0.020	0.025	− 0.001
May	− 0.091	− 0.015	− 0.065	− 0.019
June	− 0.052	− 0.019	− 0.015	0.008
July	− 0.137*	− 0.079*	− 0.069	− 0.032
Aug	− 0.092	− 0.071*	− 0.055	− 0.043
Sep	− 0.170**	− 0.086**	− 0.090	− 0.039
Oct	− 0.125*	− 0.081*	− 0.065	− 0.051
Nov	− 0.174**	− 0.072*	− 0.171**	− 0.062*
Dec	− 0.095	− 0.040	− 0.097	− 0.057*
Level 2—home block group				
Population density (× 100)	–	− 0.002***		− 0.002**
% Young males	–	0.018**		0.017**
Concentrated poverty	–	0.155		0.103
Residential instability	–	− 0.006		0.007
Racial diversity	–	− 0.388*		− 0.405**
Level 2—crime block group				
Population density (× 100)	–	–	− 0.005***	− 0.005***
% Young males	–	–	− 0.026	0.001
Concentrated poverty	–	–	− 0.086	0.035
Residential instability	–	–	− 0.013	− 0.001
Racial diversity	–	–	− 0.296	− 0.356
Constant	1.190***	2.119***	1.670***	2.435***
Model statistics				
Interclass correlation	–	0.829	0.135	–
Akaike info. criterion	6381.802	3410.211	5989.914	2566.302

*ref.* reference category, *AIAN* American Indian-Alaskan Native

*p < .05; **p < .01; ***p < .001

+Observations are nested in home block groups

++Observations are nested in crime block groups

+++Observations are cross-nested in home and crime block groups

Model 2 introduced characteristics of the individual’s home block group to the base model, and nested observations within these home block groups. The results suggest that although the residence-to-crime distance was significantly reduced during the Safer-at-Home period, the relationships appear weaker, and individual associations changed. For instance, age no longer was significantly associated with the residence-to-crime distance, whereas race remained significant. Again, individuals who identified as Black had significantly longer residence-to-crime distances than those who identified as White (*b* = − .051; p = .024). At the neighborhood level, individuals who resided in neighborhoods with higher population densities (*b* = − .00001; p = .001) and higher levels of racial diversity (*b* = − .388; p = .015) had significantly shorter residence-to-crime distances, while those living in neighborhoods with a greater percentage of young males had significantly longer residence-to-crime distances (*b* = .018; p = .002).

Model 3 introduced characteristics of the block group where the crime was committed to the model. Similar to Model 2, the associations of the Safer-at-Home order and individual characteristics were attenuated once neighborhood characteristics were entered into the model. Among these neighborhood measures, only population density of the block group where the crime occurred showed a significant association (*b* = − .00005; p < .001). Neighborhoods with higher population densities experienced crimes for which individuals lived closer to the crime. Note that the intraclass correlation is much lower when nesting observations in the crime block groups (ICC = 0.135), as opposed to nesting in the home block groups (ICC = 0.829).

Finally, Model 4 specifies that observations are cross-nested in both the individual’s home block group and the block group where the crime was committed. Again, the results support the hypothesis that the Safer-at-Home order significantly shortened the average distance between residence and crime. Specifically, the average residence-to-crime distance prior to the Safer-at-Home order was roughly 0.70 miles further (p < .001), compared to during the Safer-at-Home order [(2.435 + 0.140)^2^ −  (2.435)^2^ = 6.63–5.93)]. Similarly, residence-to crime distances were approximately 0.61 miles longer in the time following the disbandment of the Safer-at-Home order, than during the time in which the order was in place (p = .002). Both relationships were statistically significant, even when controlling for individual and neighborhood factors. On the other hand, associations between individual variables and distance to crime were smaller in Model 4 than Model 1. Lastly, neighborhood level associations, both of the home and crime block groups, remain largely unchanged from previous models.

The final series of models report the results from offense-specific models to test whether the relationship of the Safer-at-Home order, as well as all individual and neighborhood level variables were associated by crime type (Table 4). These models employed the same cross-classified model specification as used for Model 4 in Table 3. Findings showed that residence-to-crime distance was significantly longer during the pre-period than during the Safer-at-Home order for violent (*b* = .329; p = .016) and property offenses (*b* = .085; p = .030), but only significantly longer post-period than during the Stay-at-Home order for property offenses (*b* = .093; p = .027); the Safer-at-Home order was not significantly associated with residence-to-crime distance for disorder offenses. Additional file 1: Appendix provides results from supplemental analyses examining additional disaggregation by offense type, as well as effect sizes, standard errors, and p-values for all models. While the additional model findings may suggest greater clarity about which types of crime opportunities were associated with the Safter-at-Home order, we caution drawing strong conclusions when samples sizes are low (e.g., N = 54 burglaries).

**Table 4:** Factors associated with offense-specific residence-to-crime distance in midwest suburb

Variable	Violent (N = 282)	Property (N = 1552)	Disorder (N = 1092)
Level 1			
Time period (ref. = During)			
Pre-Safer-at-Home	0.392*	0.085*	0.069
Post-Safer-at-Home	0.189	0.093*	0.064
Age (ref. = age 0–17 years)			
18–24	0.180	− 0.005	− 0.003
25–34	0.079	0.018	− 0.053
35+	0.018	0.014	− 0.148**
Male (ref. = Female)	– 0.030	0.007	0.006
Race (ref. = White)			
AIAN	0.000	− 0.058	0.277
Asian	0.521	0.017	− 0.033
Black	0.103	− 0.020	− 0.086*
Month (ref. = Jan)			
Feb	− 0.209	0.047*	− 0.001
Mar	− 0.101	0.059*	− 0.053
Apr	− 0.020	0.080**	− 0.098
May	− 0.046	0.033	− 0.056
June	0.015	0.060*	0.002
July	− 0.154	0.006	− 0.103
Aug	− 0.114	0.045	− 0.136*
Sep	0.090	0.005	− 0.134*
Oct	− 0.457**	0.007	− 0.099
Nov	− 0.096	0.013	− 0.272*
Dec	− 0.233	0.030	− 0.091
Level 2—home block group			
Population density (× 100)	− 0.001	− 0.001	− 0.002**
% Young males	0.030*	0.011*	0.021**
Concentrated poverty	0.965***	− 0.092	0.339*
Residential instability	0.007	0.042	0.015
Racial diversity	0.147	− 0.438**	− 0.369
Level 2—crime block group			
Population density (× 100)	− 0.005***	− 0.005***	− 0.004***
% Young males	− 0.045*	− 0.001	0.008
Concentrated poverty	0.158	− 0.501*	0.146
Residential instability	− 0.001	0.067	− 0.027
Racial diversity	− 0.565	0.053	− 0.315
Constant	1.717***	2.308***	2.510***

*AIAN* American Indian-Alaskan Native

^*^p < .05; **p < .01; ***p < .001

Few significant individual associations emerged in the offense-specific models. Here, those who were 35 years of age or older had significantly shorter distances between their residences and the locations of their offenses, although this association only applied to disorder offenses (*b* = − .148; p = .002). Also limited to only disorder offenses, individuals identified as Black engaged in disorder offenses that were significantly closer to their residences, when compared to individuals who identified as White (*b* = − .086; p = .016).

Turning to neighborhood level associations, within home block group variables, population density was negatively associated with the distance between residence and disorder crimes (*b* = − .00002; p = .004). Across all crime type models, the percent of young males was positively associated with distance between residences and the location of the offense. Concentrated poverty was positively associated with the distance between residences and violent and disorder offenses (*b* = .339; p = .012), whereas racial diversity was negatively associated with the distance between residences and property offenses (*b* = − .438; p = .003). When considering neighborhood level associations based on the location of where the offense occurred, population density was positively associated with the distance between residence and each of the crime types. The percent of young males was negatively associated with the distance between residence and violent offenses (*b* = − .045; p = .024), whereas concentrated poverty was positively associated with the distance between residence and property offenses (*b* = − .501; p = .022).

## Discussion and conclusion

This study examined the relationship between social distancing restrictions stemming from COVID-19 (i.e., Safer-at-Home order) and the distance between an individual's home and the location where they committed a crime (i.e., residence-to-crime distance). Assuming decreased travel to nodes outside of one’s home anchor point or neighborhood during abrupt social distancing restrictions, we hypothesized that residence-to-crime distances would be shorter when a Safer-at-Home order was in place. This expectation was rooted in the observation that people are most likely to offend in their awareness space, including nodes and the pathways to those nodes (Bernasco, 2010; Bernasco & Block, 2009). Given that the Safer-at-Home order sought to reduce travel outside of the home, individuals likely did not have ample reason to travel to previously frequented nodes.

Consistent with theories in environmental criminology, we expected individuals to commit crimes closer to their homes, as these are the areas where an individual was likely to spend most of their time. In relation to prior research in urban areas, individuals from our suburban sample travelled similar or longer distances to commit crimes than their urban counterparts. Median residence-to-crime distances in our sample ranged from about 3 to 5 miles across classifications, whereas past studies report median distances closer to 1 or 2 miles in the U.S. and in other countries in urban (Leitner & Kent, 2009; Sorg, 2016; Vandeviver et al., 2015) and suburban areas (Ratcliffe, 2003). Longer median distances consistent with our findings were observed in a more recent study in Dallas, U.S. (Ackerman & Rossmo, 2015). Thus, our study adds to the literature by showing that individuals may use different spatial decision-making processes in suburbs and sprawling urban areas, such as Dallas, than in more densely populated urban places examined in other studies.

Despite relatively long residence-to-crime distances, our results largely support the hypothesis that people generally committed crimes closer to home when they were encouraged to spend less time away from home. Indeed, crimes committed during the Safer-at-Home order occurred closer to individuals’ homes, net of key individual and neighborhood characteristics. We did observe crime type differences in this relationship (see Additional file 1: Appendix for fully disaggregated results), however, which could imply that different crime opportunity structures are affected differently by social distancing changes. It could also mean that social distancing restrictions differentially impact people with different offending patterns, generating aggregate differences between crime types. Although we do observe differences by crime type, the findings from this study lend support for routine activity theory and crime pattern theory, implying that acute disruptions to routine activities and isolation from popular travel nodes will encourage crime commission closer to home. In sum, this study found that individuals committed crimes in areas that were convenient or proximal to where they are carrying out daily activities. Due to the closure of regular nodes, individuals’ awareness spaces were likely limited to the area surrounding their homes, thereby tightening the target search radius.

A widely accepted practical response that impacts an individual’s “journey to crime” is increasing police visibility in high travel areas (i.e. offender pathways) or in crime hot spots. This common response has been used by many policing agencies as a way to curb a variety of offenses including reckless driving and various property crimes (NHTSA 2022). This policy practice has become to be known as data-driven approaches to crime and safety (DDATCS). By implementing a data-driven response for high visibility traffic response and engaging in a community-oriented approach, locations have seen a significant decrease in crime (Hall & Puls, 2010). This research has shown Safer-at-Home orders impacted an individual’s regular nodes and awareness spaces. Leveraging public awareness of high-visibility traffic enforcement can impact the pathways of an offender. The most practical policy for policing agencies to implement to address a reduced “journey to crime,” is to continue to reduce that journey.

This study is not without limitations. First, our data did not permit reliable matching of individuals across crime incidents. Although research shows that repeat offending and co-offending behavior re important factors in the journey to crime (Menting et al., 2019; Xiao et al., 2018), we chose to focus our analysis on the role of place characteristics in spatial crime patterning, which have also been shown to affect decision-making (Bernasco, 2010; Bernasco & Block, 2009). Future research should further consider the interaction of individual and co-offending patterns in the journey to crime. Second, although studies using data on ambient population movement have found that residents in states with Stay-at-Home orders (such as the state in which the sample city is located) were largely adhering to these orders (for example, Badr et al., 2020; Jacobsen & Jacobsen, 2020), we were unable to test whether individuals in our sample were generally following the order. Future research should aim to examine whether individuals who were engaging in crime adhered by Safer-at-Home orders, and if so, whether these orders resulted in homes generating higher levels of activity space, while decreasing the influence of other activity nodes.

Relatedly, we recognize that individuals do not always travel from their residence when committing a crime, and therefore, our measure may not reflect one’s “journey to crime” as it is often discussed in the literature. Although past studies have shown that individuals tend to offend in close proximity to their home residence (i.e., distance decay function) (for example, Bernasco, 2006; Rengert & Lockwood, 2009; Van Daele et al., 2012), these findings continue to expand our understanding of how disruptions in routine activities tighten the distance between one’s home address and the location of their offenses. Third, the data from this study come from one mid-sized city’s police department. We are therefore unable to capture offenses that were not reported to police and caution generalizations beyond the study site due to the possible underreporting of offenses, as well as offense reporting differences that may be experienced across larger or smaller city sizes compared to the sample (Nivette et al., 2021). Lastly, low crime counts during the Safer-at-Home order may have made it more difficult to detect significant relationships, especially for certain offense types (e.g., N = 54 burglaries in our sample). While further disaggregation we explored and reported in Additional file 1: Appendix A may suggest that more disaggregated opportunity structures should be examined, we leave it to future research to consider offense-specific patterns in greater detail.

These limitations aside, our findings point to two public safety implications. First, if major changes in routine activity patterns are to be anticipated, the number of visitors into a given neighborhood may also experience changes that differ from usual patterns with corresponding increases or decreases. Awareness of changes to spatial decision-making in instances of changed patterns of activity and how that can influence who could be in the “offender pool” at a given time and location may aid in proactive crime prevention efforts. Second, programming/events related to building relationships among community members could be initiated in racially diverse neighborhoods to reduce the likelihood that local residents choose to commit crimes close-by. Although individuals tend to offend in familiar areas (i.e. their awareness space), they are also attracted to spatial enclaves with higher perceived anonymity (Ackerman & Rossmo, 2015; Bernasco, 2010; Bernasco & Block, 2009). More diverse neighborhoods may have depleted levels of social ties (Peterson & Krivo, 2010), leading to higher levels of anonymity. Community-based programming in diverse neighborhoods could help to suppress anonymity, making the local area a riskier location for crime. Future research on these issues can help communities prepare for large scale events that could impact everyday life as greatly as the COVID-19 pandemic.

## Supplementary Information

**Additional file 1: Appendix.** Additional model results.

## Data Availability

Not applicable.

## Footnotes

1 Eleven cases were omitted due to missing home address data (i.e., individuals with unknown addresses, individuals identified to be homeless, incomplete addresses). Seven cases were omitted for missing offender demographic data. In addition, five cases were omitted because the residence-to-crime distance was greater than 20 miles.

2 Principal components factor analysis was used to create an index of concentrated disadvantage using four relevant measures (e.g., percent in poverty, % receiving public assistance). Replacing the concentrated poverty measure with this disadvantage measure did not change substantive findings.

3 Offenses occurring between January 1, 2018 and March 24, 2020.

4 Offenses occurring between March 25, 2020 and May 13, 2020.

5 Offenses occurring between May 14, 2020 and December 31, 2020.

6 Offenses were classified based on the Uniform Crime Report’s “Hierarchy Rule,” in which incidents that included numerous offenses were coded as the offense of the highest severity.

## References

[CR1] Ackerman JM, Rossmo DK (2015). How far to travel? A multilevel analysis of the residence-to-crime distance. Journal of Quantitative Criminology.

[CR2] Andresen MA, Frank R, Felson M (2014). Age and the distance to crime. Criminology & Criminal Justice.

[CR3] Badr HS, Du H, Marshall M, Dong E, Squire MM, Gardner LM (2020). Association between mobility patterns and COVID-19 transmission in the USA: A mathematical modelling study. The Lancet Infectious Diseases.

[CR4] Bernasco W (2006). Co-offending and the choice of target areas in burglary. Journal of Investigative Psychology and Offender Profiling.

[CR5] Bernasco W (2010). A sentimental journey to crime: Effects of residential history on crime location choice. Criminology.

[CR6] Bernasco W, Block R (2009). Where offenders choose to attack: A discrete choice model of robberies in Chicago. Criminology.

[CR7] Bernasco W, Nieuwbeerta P (2005). How do residential burglars select target areas? A new approach to the analysis of criminal location choice. British Journal of Criminology.

[CR8] Blau PM (1977). Inequality and heterogeneity: A primitive theory of social structure.

[CR9] Boivin R, Felson M (2018). Crimes by visitors versus crimes by residents: The influence of visitor inflows. Journal of Quantitative Criminology.

[CR10] Brantingham PJ, Brantingham PL (1981). Environmental criminology.

[CR11] Brantingham PL, Brantingham PJ (1993). Nodes, paths and edges: Considerations on the complexity of crime and the physical environment. Journal of Environmental Psychology.

[CR12] Browning CR, Pinchak NP, Calder CA (2021). Human mobility and crime: Theoretical approaches and novel data collection strategies. Annual Review of Criminology.

[CR13] Bursik RJ, Grasmick HG (1993). Economic deprivation and neighborhood crime rates, 1960–1980. Law & Society Review.

[CR14] Cahill M, Mulligan G (2007). Using geographically weighted regression to explore local crime patterns. Social Science Computer Review.

[CR15] Campedelli GM, Favarin S, Aziani A, Piquero AR (2020). Disentangling community-level changes in crime trends during the COVID-19 pandemic in Chicago. Crime Science.

[CR16] Castle YA, Kovacs JM (2021). Identifying seasonal spatial patterns of crime in a small northern city. Crime Science.

[CR17] Ceccato V, Kahn T, Herrmann C, Östlund A (2022). Pandemic restrictions and spatiotemporal crime patterns in New York, São Paulo, and Stockholm. Journal of Contemporary Criminal Justice.

[CR18] Clarke RV, Cornish DB (1985). Modeling offenders’ decisions: A framework for research and policy. Crime and Justice.

[CR19] Cohen LE, Felson M (1979). Social change and crime rate trends: A routine activity approach. American Sociological Review.

[CR20] Curtis-Ham S, Bernasco W, Medvedev ON, Polaschek D (2020). A framework for estimating crime location choice based on awareness space. Crime Science.

[CR21] Elffers H, Reynald DM, Averdijk M, Bernasco W, Block R (2008). Modelling crime flow between neighbourhoods in terms of distance and of intervening opportunities. Crime Prevention and Community Safety.

[CR22] Felson M, Jiang S, Xu Y (2020). Routine activity effects of the COVID-19 pandemic on burglary in Detroit, March 2020. Crime Science.

[CR23] Halford E, Dixon A, Farrell G, Malleson N, Tilley N (2020). Crime and coronavirus: Social distancing, lockdown and the mobility elasticity of crime. Crime Science.

[CR101] Hall, H., & Puls, E. N. (2010). Implementing DDACTS in Baltimore County: Using geographic incident patterns to deploy enforcement. *Geography & Public Safety,**2*(3), 5–7.

[CR24] Hammond L, Youngs DE (2011). Decay functions and offender spatial processes. Journal of Investigative Psychology and Offender Profiling.

[CR25] Jacobsen GD, Jacobsen KH (2020). Statewide COVID-19 Stay-at-Home Orders and population mobility in the United States. World Medical & Health Policy.

[CR26] Johnson BD (2012). Cross-classified multilevel models: An application to the criminal case processing of indicted terrorists. Journal of Quantitative Criminology.

[CR27] Lammers M (2018). Co-offenders’ crime location choice: Do co-offending groups commit crimes in their shared awareness space?. The British Journal of Criminology.

[CR102] Leitner, M., & Kent, J. (2009). Bayesian journey-to-crime modelling of single and multiple crime-type series in Baltimore County, MD. *Journal of Investigative Psychology and Offender Profiling,**6*(3), 213–236.

[CR28] Massey D, Booth A, Crouter AC (2001). Does it take a village? Community effects on children, adolescents, and families. The prodigal paradigm returns: Ecology comes back to sociology.

[CR29] Menting B, Lammers M, Ruiter S, Bernasco W (2019). The influence of activity space and visiting frequency on crime location choice: Findings from an online self-report survey. The British Journal of Criminology.

[CR30] Mohler G, Bertozzi AL, Carter J, Short MB, Sledge D, Tita GE, Uchida CD, Brantingham PJ (2020). Impact of social distancing during COVID-19 pandemic on crime in Los Angeles and Indianapolis. Journal of Criminal Justice.

[CR103] National Highway Traffic Safety Administration (NHTSA). (2022). High visibility enforcement (HVE) toolkit. Accessed online at https://www.nhtsa.gov/enforcement-justice-services/high-visibility-enforcement-hve-toolkit#placement-32156.

[CR31] Nivette AE, Zahnow R, Aguilar R, Ahven A, Amram S, Ariel B, Burbano MJA, Astolfi R, Baier D, Bark H-M, Beijers JEH, Bergman M, Breetzke G, Concha-Eastman IA, Curtis-Ham S, Davenport R, Díaz C, Fleitas D, Gerell M, Jang K-H, Kääriäinen J, Lappi-Seppälä T, Lim W-S, Revilla RL, Mazerolle L, Meško G, Pereda N, Peres MFT, Poblete-Cazenave R, Rose S, Svensson R, Trajtenberg N, van der Lippe T, Veldkamp J, Vilalta Perdomo CJ, Eisner MP (2021). A global analysis of the impact of COVID-19 stay-at-home restrictions on crime. Nature Human Behaviour.

[CR32] O’Leary M (2011). Modeling criminal distance decay. Cityscape.

[CR33] Peterson RD, Krivo LJ (2010). Divergent social worlds: Neighborhood crime and the racial-spatial divide.

[CR34] Peterson RD, Krivo LJ, Harris MA (2000). Disadvantage and neighborhood violent crime: Do local institutions matter?. Journal of Research in Crime and Delinquency.

[CR104] Ratcliffe, J. H. (2003). Suburb boundaries and residential burglars. No 246. *Trends and Issues in Crime and Criminal Justice*. Australian Institute of Criminology.

[CR35] Raudenbush SW, Bryk AS (2002). Hierarchical linear models: Applications and data analysis methods.

[CR36] Rengert GF (2002). The journey to crime.

[CR37] Rengert GF, Lockwood B (2009). Geographical units of analysis and the analysis of crime. Putting crime in its place.

[CR38] Sampson RJ, Groves WB (1989). Community structure and crime: Testing social-disorganization theory. American Journal of Sociology.

[CR39] Sampson RJ, Morenoff JD, Gannon-Rowley T (2002). Assessing “neighborhood effects”: Social processes and new directions in research. Annual Review of Sociology.

[CR40] Shaw CR, McKay HD (1942). Juvenile delinquency and urban areas.

[CR41] Simpson EH (1949). Measurement of diversity. Nature.

[CR42] Song G, Bernasco W, Liu L, Xiao L, Zhou S, Liao W (2019). Crime feeds on legal activities: Daily mobility flows help to explain thieves’ target location choices. Journal of Quantitative Criminology.

[CR43] Sorg ET (2016). Classifying import and domestic hot spots of crime by offender home proximity. Policing: A Journal of Policy and Practice.

[CR45] United States Census Bureau. (2019). *American Community Survey*. Retrieved from www.census.gov/acs/www/data/data-tables-and-tools/data-profiles/2019a/.

[CR46] Van Daele S, Cools M, De Ruyver B, Easton M, Pauwels L, Ponsaers P, Van de Walle G, Van der Beken T, Van der Laenen F, Vermeulen G, Vynckier G (2010). Mobility and distance decay at the aggregated and individual level. Safety, societal problems and citizens’ perceptions: New empirical data, theories and analyses.

[CR47] Van Daele S, Vander Beken T, Bruinsma GJN (2012). Does the mobility of foreign offenders fit the general pattern of mobility?. European Journal of Criminology.

[CR48] Vandeviver C, Neirynck E, Bernasco W (2021). The foraging perspective in criminology: A review of research literature. European Journal of Criminology.

[CR49] Vandeviver C, Van Daele S, Vander Beken T (2015). What makes long crime trips worth undertaking? Balancing costs and benefits in burglars’ journey to crime. British Journal of Criminology.

[CR50] Xiao L, Liu L, Song G, Ruiter S, Zhou S (2018). Journey-to-crime distances of residential burglars in China disentangled: Origin and destination effects. ISPRS International Journal of Geo-Information.

[CR51] Yang M, Chen Z, Zhou M, Liang X, Ziyue B (2021). The impact of COVID-19 on crime: A spatial temporal analysis in Chicago. ISPRS International Journal of Geo-Information.

